# The Recent Vaccination in Atlanta

**Published:** 1882-08

**Authors:** John H. Logan

**Affiliations:** Atlanta, Ga., Professor of Chemistry in the Atlanta Medical College


### THE RECENT VACCINATION IN ATLANTA.

### HISTORY OF THE MOVEMENT—ITS METHODS, RESULTS, CONCURRENT FACTS, AND THE RELATIONS OF THESE TO SANITARY MEDICINE AND SOCIAL LIFE.

BY JOHN H. LOGAN, M.D., ATLANTA, GA.,
Professor of Chemistry in the Atlanta Medical College.

Early in the autumn of 1881, variola made its appearance and prevailed with noticeable malignancy in several of the principal cities of the North and Northwest, soon after the great cotton exposition of Atlanta was at the full tide of its success, with an immense concourse of people of all ages and from every part of the Union, coming and going. Under these circumstances the guardians of our city's health, full of apprehension for the safety of the people and their commercial prosperity, resolved, with the concurrence of a wide-awake city council, to proceed at once to put the entire community, as far as possible, under the protective influence of vaccination.

It would be doing great injustice, however, to the gentlemen of the Board of Health to leave the impression that they were duly moved to act at this time by the threatened advent of a dangerous epidemic. Long before this date they had been actively engaged with all the light, and doubtless with all the power then at their command, to set the city in order against the possible invasion of small-pox.

The history of this movement is not a little interesting, and may be divided into three distinct acts or stages. The first relates exclusively to the great public school system of Atlanta. As far back as February, 1880, the following communication was addressed by the Secretary of the Board of Health, Dr. Jas. B. Baird, to the Superintendent of the Public Schools:

"Dear Sir: I would hereby call your attention to the importance of enforcing at this time the rule requiring vaccination in the public schools of Atlanta, as small-pox is prevailing in some of the Northern and Western cities."

At the following meeting of the Board of Education, February 28th, this communication from the Secretary of the Board of Health was submitted by the Superintendent, together with suggestive information furnished from the North relating to the prevalence of variola in cities and public schools there.

It was then that Mr. David Mayer, with his usual promptness in such matters, moved that the sixty-fourth rule in the government of the public schools be rigidly enforced—the latter clause of which reads thus: "nor shall any pupil be admitted to any public school without giving satisfactory evidence of having been vaccinated."

This motion was unanimously sustained, and fairly inaugurated the first stage of that series of acts by which Atlanta has come to be the most thoroughly vaccinated city in the United States. At the same meeting it was also resolved that the children be allowed till the 1st of April to give satisfactory evidence of vaccination, with instructions to the Superintendent to suspend all from that date who failed to comply. This was pushing matters a little too fast, however, for there were obstacles in the way of the complete and speedy accomplishment of the praiseworthy design of the Board, of which they were at that time ignorant. They had made no estimate of the general popular prejudice against the anti-variolous process of Jenner—a prejudice not at all confined to illiterate whites and blacks, but cropping out from many educated and even professional sources. This fact is remarkable, but nevertheless true, as was often verified in the history of this vaccination movement. They had made no proper estimate of the time and cost of effectually vaccinating hundreds of persons so diversely conditioned in social life. They had no idea of the difficulty and cost of procuring a sufficient supply of reliable vaccine virus. At that time, little or none could be got from the State vaccine agent, and no provision had been made to furnish it from the bovine lymph farms. It may be fairly said, too, that no blame was due for this deficiency to either the Governor or the State vaccine agent. Even the bovine lymph farms, at that juncture, could scarcely supply as reliable lymph as that which they have since been able to prepare and transport. They are constantly improving their method of conserving the efficiency of the vaccine germ, but even yet the process is but in the infancy of its development.

Irregularity and slow progress in the work necessarily became manifest. Some refused to be vaccinated at all; many were too poor to meet the cost, while others were pronounced by family physicians to be in a physical condition that rendered vaccination dangerous.

Any physician or amateur operator was authorized to vaccinate; it was only required that it should be done effectually, and so certified.

What was to be done? The Board of Education met again on the 25th of March. The Superintendent submitted a report, one statement of which greatly surprised all present; there were many connected with the schools who manifested opposition to the practice of vaccination.

Our guardians of education proved themselves equal to the emergency. The resolution of Mr. Mayer reiterated with a meaning emphasis, and a strong committee was appointed to see that neither the resolution nor the public schools should suffer detriment, and determined that henceforth the whole matter of vaccination should be referred to this committee, comprised of Drs. Roach, Powell, Spaulding and Mayor English.

During the following months of April and May, the work went slowly on, irregularly and often with doubtful results from causes already detailed.

There were two of the schools in which the difficulties noted were particularly inherent. These were the colored schools of Summer Hill and Hayne street. In these schools at that time, there were no less than four hundred unvaccinated pupils. The greater part of these were practically unable to pay the cost of their vaccination, even at a low figure, the operator, of course, being compelled to supply the lymp, and little or none procurable from the State agent. Meantime, to complicate matters, the successive circulars of the superintendent were one after the other, appearing to urge up the teachers to the duty of attending personally to this work; the first previous to the 1st of April, 1880, and the last preceding the 1st of January, 1881.

The lady principals, presiding in these schools, Mrs. Mercer and Mrs. Harvey, representing two well-known families in Georgia, the former having lost her husband in the army of Virginia, and a once fine estate in the general wreck of the war, were almost in despair. They felt the responsibility laid upon them, but how to discharge it practically was a serious question.

For some time squads of the children from Hayne street school had been sent daily, nearly a mile across the city to be vaccinated at a clinic held by several young physicians in the Atlanta Medical College. But this was slow work, and caused confusion and irregularity in the daily routine of the school. At this juncture, Mrs. Mercer, the principal, sent for the writer and begged him to help her out of her dilemma.

It was then, that I for the first time, got an insight into the difficulties of attempting to vaccinate, even for their own good, a whole community of people. The request of a lady could not be declined. From Dr. Robert Westmoreland, the State vaccine agent, I obtained a minute fraction of a crust, and with this began vaccinating daily, first in Hayne street school, and afterwards in it and Summer Hill school alternately, charging only the small fee that had been fixed by the young men at the College clinic. For this I was severely criticised at the time, but feeling conscious of doing good work, I cared little for the censure of men, who knew nothing of, and could have no proper appreciation of my motives. All is well that ends well.

The virus I had got from Dr. Westmoreland proved unexceptionably good, and with this, and a small additional crust obtained from Dr. J. S. Todd, I succeeded long before the last day of December, 1881, in imprinting for all time, a characteristic scar upon an arm of every unvaccinated child in those colored schools.

I do not assume this result without adequate evidence; while making my last re-vaccinations—for it was necessary to visit the schools often for this purpose—the committee had deputed the city physicians to inspect the pupils of all the schools and report their condition in regard to vaccination. Their report was published soon after in the Constitution, through the Superintendent, and reads in substance as follows: "With the exception of some thirty or forty who appear to be insusceptible of the vaccine disease, every child, white and colored, of the public schools of Atlanta, is effectually vaccinated."

This *was* a great work for whose achievement the Board of Health, the Board of Education and the excellent Superintendent of the public schools deserve the lasting remembrance of our people. My reward for the subordinate part that I did in it was not money nor even a hint of public recognition, but the gratitude of the principals of the two colored schools, and the consciousness of having done a good work for those who could not help themselves. At this hour there is not, in the world, a more thoroughly vaccinated system of public schools than those of our city. And now for all time to come, the Superintendent holds the situation in his own hands, for the great difficulty overcome by the Board was to effect the vaccination of the teeming numbers who had already entered the shools without the safeguard of the vaccine cicatrix. Will the Board of Health secure the same vantage ground in regard to the future of the entire city? But this is only part first of the history of vaccination in Atlanta. I now enter upon the second.

It has already been remarked that during the Cotton Exposition, when our city became the temporary home of thousands of strangers from every part of the country, many of them from infected cities, an hourly invasion by small-pox was anticipated by the watchful Board of Health. Prompted thus, they determined to meet the invader more than half way. At a meeting on the 27th of December, 1881, a few days before the close of the Exposition, it was resolved with the co-operation of the city council to appoint five vaccinators and a supervisor, equip them with fresh bovine lymph and put them to work, one in each of the five city wards. It was also provided that as soon as a supply of humanized virus could be procured from the primary vaccinations already soon to be made, two central offices for free vaccination, one for the whites and the other for negroes, should be opened in different parts of the city. Dr. W. H. Cumming was put at the head of this corps of vaccinators, and they went to work with carefully selected lymph on points procured from Messrs. Codman & Shurtleff, Boston, Mass., and John Wyeth & Brother, Philadelphia.

On examination, after due time for incubation, it was discovered that scarcely 25 per cent. of the vaccinations had proved effective. Why was this? Was the lymph defective in that proportion, or was it attributable to the method of vaccination? This is a practical question that I know now to be of no little importance. I observed that most of the vaccinators used the lancet; a few the needle or scarificator; some merely punctured the arm in several points, while others scarified with as little blood as possible in the same number of points. It had been agreed, too, at the outset, that no more than two persons should be vaccinated from the same lymph point.

It became manifest that the lancet with the punctures was the more efficient instrument. But this was only comparative. I have reason now to teach that neither the lancet with the punctures, nor the bloodless scarification with the needle is the best method of vaccination. A single lymph point, too, to two persons is bad practice. No operator will regret, even when using the best bovine lymph, to give an entire point to a single person, or even two, as I have frequently done, when it was more important to secure a successful result than to practice a doubtful economy. As regards the manner of the scarification, it was found always best and surest to execute this with a good lancet in a single space as large as a dime, or better, a nickel, by parallel vertical cuts, crossed by as many horizontal cuts, made so deep as to draw blood. I repeat, so as to draw blood.

I am aware that some, at least, of the bovine farmers emphasize just the contrary injunction, "Draw no blood." But I am convinced that if they had ever tested the matter, as has recently been done in Atlanta, they would have withheld from their envelopes advice so adverse to their interest. First, it is all bosh to say that the oozing blood washes out the lymph from the wound. I have witnessed the contrary a thousand times. So far from that being true, the presence of the oozing blood, I contend, is the most favorable physiological condition for the absorption of the lymph. All zymotic germs, such as vaccine lymph and the virus of the rattlesnake, find in the welling blood of a wound their most genial medium for rapid diffusion. They diffuse more rapidly along the current of the outflowing blood towards the bottom of the wound than the blood moves towards the outlet of the wound. Dame Nature makes no mistakes. If any one has any doubts on this point, let him examine the fangs of the rattlesnake and believe. Do they appear as if designed simply to scrape through the epidermis to the true skin without drawing blood? Its sting never fails to draw blood, and the deadly innoculation of its virus never fails of its mark. On this question of drawing blood in vaccination, I quote the following from Reynold's System of Medicine. Speaking of the several methods of vaccinating, the writer says: "Any fear that the bleeding which ensues will cause the vaccination to fail is quite chimerical." Suppose, for argument's sake, that the fangs of the rattlesnake were charged with vaccine lymph just before his fatal blow instead of their own peculiar virus, would he not be a most successful vaccinator, as he is the most unfailing innoculator, using the latter word in in its common and not technical sense? The law by which the vaccine lymph, bearing its specific germ is diffused through the blood, and then and nowhere else, incubates, is yet beyond the pale of our knowledge. We only know that the warm living blood is its recipient.

Only about 25 per cent. of successful vaccinations, falling so far short of the high standard in England—not above one failure in 150 times—was rather discouraging. But much valuable experience—some of which has just been detailed—was gleaned from our effort, and enough of successful cases found to establish the central offices. These were promptly opened, one on Whitehall street for negroes, and the other at the City Hall for whites, early in January, 1881, and kept daily in operation, Sundays excepted, for about nine weeks.

In the first week of the work from house to house 1,126 persons received the virus, and from these the humanized lymph was obtained by which the work was continued in the central offices. And in nine weeks from the 27th of December, 1881, embracing 118 persons vaccinated at the Atlanta University, 170 in Storrs School, and 35 in the Gate City School, there were 1,769 whites and 3,123 negroes vaccinated, making a sum total of 4,892 persons. The aggregate cost of this, in money expended for salaries, virus, office rent, coal and incidentals, was $731.45, or a little over 15 cents per capita.

Early in March the central offices were closed, no more persons presenting themselves for vaccination, and no invasion of the dreaded disease having taken place. The small-pox scare was said to be at an end. And thus closed the second stage of the history of vaccination in Atlanta.

All was quiet along the Chattahoochee. The daily reports of small-pox in Chicago, Cincinnati, and even in Nashville and Chattanooga had almost ceased to attract attention. The city deemed herself quite secure, having vaccinated nearly 5,000 of her unprotected citizens, and nearly every pupil in her public schools hopefully scared by vaccination to begin with, besides the large numbers of the people who had been long ago or more recently vaccinated in private offices or by the family physician. But it is the unexpected that always happens. The city had not sufficiently estimated her danger from epidemics lurking in her large, vagrant negro population.

April 3d, 1882, dissipated the dream of exemption from small-pox. That morning it was announced that one Myra Tate, colored, had been found at a dirty negro resort, known as the "Beaver Slide," on Ivy street, sick with confluent variola. There could be no doubt about it; experienced physicians had examined the case and so pronounced it. April 16th another case is reported at the residence of Capt. Foute, of the city police, on Whitehall street; another in a different place on the 17th, and then cases quickly follow on the 18th, 20th, 21st and 22d of the same month, all negroes. The city was aroused. Assafœtida nearly doubled in price, and crinoline suddenly ceased to flash and flutter in the vicinity of the infected points. The Board of Health came together at the first intimation of danger, and without loss of time both they and the city council were concerting plans characteristic of Atlanta energy to stamp out the invading disease. Bovine lymph, isolation and fire were the prime means voted for defense. The Beaver Slide was reduced to ashes, the rising flames being held back from neighboring buildings by watchful firemen. Beds and clothing of the sick were consumed by fire, while the patients themselves were quickly removed in close covered ambulances to the hospital provided for them outside of the city. But all this was only preliminary to the great work to be achieved; the Board of Health, supported by the equally energetic city council, resolved that the entire city should be thoroughly vaccinated. The presence on the body of scars, however pearly and pitted, was to be no guaranty of safety; vaccination and revaccination was the order of the day. Bovine lymph was brought in by the package from Boston and Philadelphia, from rural farms in Pennsylvania, the District of Columbia, and even from Louisiana.

An office for free vaccination was established up stairs in the central police station for whites and blacks, and an office was also opened in each ward of the city, while thousands of circulars scattered broadcast over the city called upon the people to go, with their children and servants to be vaccinated. In addition to this a vaccinating officer was placed in each ward and ordered to vaccinate from house to house, while a corps of sanitary inspectors, with a mounted physician at their head, were detailed from the police force, whose duty it was to scour the city in search of new cases, and when discovered, to remove or quarantine them as quickly as possible. The Board of Health, as represented by their president and secretary, Drs. Armstrong and Baird, held a levee every morning to receive reports, direct the work and distribute the daily supply of vaccine lymph.

More than one thousand people, men, women and children, pressed into the room of the central vaccinator the day of its opening; and this excitement continued for days. Every one was vaccinated who came, irrespective of old vaccine scars or the repeated assertions of many that they had already experienced small-pox or varioloid. There were instances where, notwithstanding the obvious presence of pox-marks, the parties received vaccination, and in due time developed all the symptoms and marks of vaccinia. This often occurred in persons who, having no marks of variola, yet claimed to have passed through varioloid; for some of these were lying to avoid vaccination, while a larger number believed honestly that they had suffered varioloid, when it was only varicella, or chicken-pox. It was observed, however, by all the vaccinators, as important to the claim of Jenner for the protective influence of vaccinia, both against variola and its own repetition, that there was scarcely one per cent. of those who were well marked by vaccine scars, either adults or children, who were found to be again susceptible of that disease. This was attested again and again. There were found a very few persons out of whose systems the protective powers of vaccinia appears to have faded, from some utterly unknown cause. But the popular notion that it is lost at the end of seven years, or wears out of the system in that time is a vulgar delusion. No well informed physician could entertain such an opinion. Why some persons are susceptible of measles, or small-pox, or vaccinia, after the first attack, has never been explained satisfactorily.

Among those vaccinated at the central police station were many foreigners, who had been vaccinated in Europe, and the work was well done, if the number and characteristic features of the scars were any indication. The recently arrived Russian Jews presented the best examples, being beautifully scarred, as a vaccinator would say, on both arms, generally two or three large, pitted cicatrices were displayed on each. Several of these came to me again and again, with their children to be re-vaccinated, especially after the publication of the compulsory ordinance, appearing anxious to comply with every form of law. In no instance were their re-vaccinations successful, and at last, I had to tell them that I would not repeat it again. I was particularly struck with the fine eyes and physical beauty of their children and wondered how the Russians could hate and oppress them. They will find a happy home in the Southern United States.

The vaccinators, who were now at work, and who had also labored last winter in the same field, could not but be struck with the far greater success they were now having with bovine lymph transported in the same manner from the same farms. Scarcely a single primary vaccination failed. Those done at the central police station, notwithstanding the torrents of blood reported to have been shed there, were remarkably successful. Some of the negroes complained that they were almost cut to pieces in the operation and afterwards nearly died from the vaccine disease that came from the slashing and cutting!

Why was this greater success, now, than last winter? Was it due to the use of better lymph? The same kind of lymph was used in both instances. Was it due to the improved method of vaccination? I believe it was, and have given my reasons for it in another place. The truth is, I once thought that anyone could perform vaccination, I am not of that opinion now. There is in it more of science than sleight of hand, and the sure immunity it offers, when well and properly done, from a dangerous and loathsome disease, attaches to it a responsibility too heavy for the unskilled, and far beyond the comprehension of the ignorant. This remark is in keeping with the teaching of Jenner and of all experienced vaccinators. The liability of any individual to have small-pox severely after vaccination, and probably the liability to take it at all will be inversely as the thoroughness of the vaccination.* Jenner, himself, says: "Duly and efficiently performed, it will protect the constitution from subsequent attacks of small-pox as much as that disease itself will. I never expected it would do more, and it will not, I believe, do less. But mark, it must be properly and efficiently done; then, it must be allowed to run a regular course, giving the surest evidence of infecting the constitution."

By this time the greater part of the population had been vaccinated, and thousands re-vaccinated again and again. But it now became apparent that there was in the city a large class of both white and colored persons who, for various reasons, refused to be vaccinated. This necessarily effectually blocked the City Council and Board of Health in their design to effect the thorough vaccination of the entire city. What was to be done?

At this juncture the following ordinance submitted by

*Reynold's System of Medicine.

the Board of Health was presented and unanimously adopted:

WHEREAS, The disease known as small-pox has assumed such proportions in the city of Atlanta as to require the earnest and immediate attention of the city authorities; and whereas, it is the opinion of the medical profession of the world, and an established fact in medical science, that thorough vaccination is an absolute preventive of this disease; and whereas, the Board of Health of this city have, after careful consideration of the subject, recommended to this body the passage of an ordinance providing for an immediate and effective vaccination of of all residents of the city.

Now, in pursuance of the power vested in this body to pass ordinances it may deem proper for the health of the city, the mayor and general council of the city of Atlanta do ordain:

SECTION 1. Each and every resident in the city of Atlanta is hereby required to be at once successfully vaccinated or to be vaccinated a sufficient number of times to make it evident that successful vaccination is impossible.

Section 2. The Board of Health are hereby required to open, and keep open so long as the same may be in their judgment necessary, free vaccination offices in different parts of the city, and to provide a sufficient number of competent physicians and an ample supply of pure vaccine virus for such offices. Said Board is also directed to send competent physicians from house to house through the city to vaccinate all who have not been successfully vaccinated.

Section 3. Any person in the city of Atlanta who shall fail to comply with the requirements of the above ordinance by the 20th day of May, 1882, may be removed by the Board of Health to a suitable quarantine camp to be provided for that purpose beyond the city limits.

Section 4. Any person over fifteen years of age resident in the city of Atlanta, who shall refuse to be successfully vaccinated by the 20th day of May, 1882, shall be arrested by any officer or member of the police force and taken before the recorder's court, and on conviction thereof shall be fined in a sum not exceeding $500, or be imprisoned not exceeding thirty days, either or both, in the discretion of the court.

Section 5. Any parent, guardian, or other person having in their charge and control a child or children under fifteen years of age, who shall refuse or fail by the 20th day of May, 1882, to have such child or children vaccinated (if they have not been already successfully vaccinated), shall be arrested by any officer or member of the police force and taken before the recorder's court and shall, on conviction of such refusal or failure, be fined in a sum not exceeding five hundred dollars, or be imprisoned not exceeding thirty days, either or both, in the discretion of the court.

Section 6. Any practicing physician or other person who shall know of the existence of a case of small pox or varioloid in the city of Atlanta, and who shall fail within six hours to report the same to the Board of Health or other health officer of the city, shall be arrested by any officer or member of the police force and taken before the recorder's court, and shall on conviction be fined in a sum not less than twenty-five dollars, nor more than five hundred dollars, or be imprisoned not more than thirty days, either or both in the discretion of the court.

Section 7. Any person who shall in any way hinder or obstruct any member of the Board of Health, members of the police or sanitary force, physcian or other person from removing to the small-pox hospital or quarantine camp any person whom they desire to remove, or who shall in any way hinder, obstruct or prevent the enforcement of any of the provisions of this ordinance shall be arrested and taken by any officer or member of the police force before the recorder's court, and such person shall, on conviction, be punished as provided in the preceding section.

Section 8. The Board of Health shall provide suitable rules and regulations for the effectual carrying out of the provisions of this ordinance, and all ordinances and parts of ordinances conflicting herewith are hereby repealed.

Thus, for the first time in the history of Georgia, vaccination is enforced by law, even, by a municipal authority.

The daily order of procedure, is considerably changed. The agents of vaccination go out, now, two and two, a vaccinator armed with lancet and lymph and a policeman with his btona and a roll of blank forms of summons. The work moves on with a new impulse. The number of primary vaccinations doubles at the central office; exciting scenes are enacted in the wards; the young vaccinators turn over a whole chapter of knowledge in human nature in a single week; their vocabulâry of English, strong, but not altogether undefiled is largely augmented. Case after case goes up to the Recorder's court, and is quickly followed by a fine and a public vaccination of the contestant. These are chiefly from the colored side; but, now and then, a white recusant goes up, of some intelligence and a larger fund of pug-dog obstinancy; among them, one or two, of that well represented class in Atlanta, known as grip-sack and cure-all practitioners.

The opposers of the ordinance, generally, got the worst of it, but not in every instance, even, in the Recorder's court, for in one case a round-headed Englishman, who had enough money to brace up his obstinacy, carried his case, by way of test, up to a higher tribunal, where it is now pending. And on the strength of this protest, I hear that several young lawyers are preparing suits of damages against the city, for injuries sustained by the arms and lascerated dignity of numerous contestants.

It was amusing to hear, at this time, the leading objections to vaccination, especially by the negroes. The white folks they said were putting poison in their arms, so as to kill them off; the arms of children were rotting off in every part of the city, and many, both children and adults, had already died from vaccination, and so on. The grip-sacks came to the front and assured the negroes that there was no small-pox in the city, and never had been; the reported cases of disease were nothing more than "black measles." The negroes do not cease to wonder, today, that the small pox attacked them and not the whites.

The work, however, went bravely on and thus closed the third act of this vaccination movement in Atlanta.

It only remains to gather up the important lessons it teaches :

1. The cost to the city of the first stage, was, $1,011.45 ; of the last over $8,000.00. No estimate is made of the loss from stoppage of trade.

2. A worthless, vagrant negro, exposed to some dangerous epidemic disease, is a costly addition to any population.

3. The congregating of large numbers of idle negroes in a city, is neither good for the negroes nor for the city.

4. Idle, vicious negroes, and abortion doctors of any color or sex should not be suffered to remain in the city a single hour.

5. The best protection against small-pox is prompt and thorough vaccination.

6. In order to complete the work of vaccination, a compulsory act will be necessary.

7. Resistance to this, will be in proportion to the ignorance and grip-sack quackery that pervade the community.

8. Some permanent arrangement for vaccination, should be made, compulsory if necessary, else the city, in a few years, will be in the same exposed condition as before.

9. Bovine and humanized lymph can be preserved active for years in hermetically sealed tubes.

10. Bovine lymph, well preserved is good and though costly, must often be the only resort, till the superior method of vaccination from arm to arm can be used.

11. The most successful and protective process of vaccination is one, large, checked scarification, made with the lancet over an area the size of a nickle, down to the oozing blood of the cutis vera. The blood and lymph should be allowed to dry upon the scarification.

12. Among the thousands vaccinated in the city, there was not a single authenticated instance of death or loss of an arm from the operation.

13. Of the thousands of public school children, many of them greatly exposed to the contagion, not one suffered an attack of variola in any form.

14. During this small-pox invasion there were treated in the city and at the hospital 112 cases—of these 45 died; a little over 41 per cent. The usual mortality taken at all ages in the hospitals, is 50 per cent. for confluent small-pox; 8 per cent. for semi-confluent and 4 per cent. for the milder forms of the disease.

Dr. Nathan O. Harris was the physician in charge of the small-pox hospital and deserves great credit for the energetic, selfsacrificing spirit with which he discharged his duty.

